# Disease-related disability burden: a comparison of seven chronic conditions in middle-aged and older adults

**DOI:** 10.1186/s12877-021-02137-6

**Published:** 2021-03-23

**Authors:** Chieh-Ying Chou, Ching-Ju Chiu, Chia-Ming Chang, Chih-Hsing Wu, Feng-Hwa Lu, Jin-Shang Wu, Yi-Ching Yang

**Affiliations:** 1 Institute of Gerontology, College of Medicine, National Cheng Kung University, No. 1, University Road, 70101 Tainan, Taiwan; 2 Department of Family Medicine, National Cheng Kung University Hospital, Tainan, Taiwan; 3 Department of Medicine, College of Medicine, National Cheng Kung University, Tainan, Taiwan; 4 Division of Geriatrics and Gerontology, Department of Internal Medicine, National Cheng Kung University Hospital, Tainan, Taiwan; 5 Department of Family Medicine, College of Medicine, National Cheng Kung University, Tainan, Taiwan

**Keywords:** Physical disability, Trajectory, Chronic condition, Middle-aged and older Taiwanese

## Abstract

**Background:**

Although previous studies have explored the effect of chronic conditions on physical disability, little is known about the levels and rates of change in physical disability after a chronic condition diagnosis in middle-aged and older adults in the Asian population. The aim of this study is to ascertain the average levels and rates of change in the development of disability after disease diagnosis, as well as to determine the influences of sociodemographic and health-related correlates in the development of disability.

**Methods:**

This is a retrospective cohort study analyzing data of nationally representative participants aged 50 and over with a chronic condition or having developed one during follow-ups based on data from the 1996–2011 Taiwan Longitudinal Study on Aging (TLSA) (*n* = 5131). Seven chronic conditions were examined. Covariates included age at initial diagnosis, gender, education level, number of comorbidities, and depression status. Physical disability was measured by combining self-reported ADL, IADL, and strength and mobility activities with 17 total possible points, further analyzed with multilevel modeling.

**Results:**

The results showed that (1) physical disability was highest for stroke, followed by cancer and diabetes at the time of the initial disease diagnosis. (2) The linear rate of change was highest for stroke, followed by lung disease and heart disease, indicating that these diseases led to higher steady increases in physical disability after the disease diagnosis. (3) The quadratic rate of change was highest in diabetes, followed by cancer and hypertension, indicating that these diseases had led to higher increments of physical disability in later stage disease. After controlling for sociodemographic and comorbidity, depression status accounted for 39.9–73.6% and 37.9–100% of the variances in the physical disability intercept and change over time, respectively.

**Conclusions:**

Despite the fact that a comparison across conditions was not statistically tested, an accelerated increase in physical disabilities was found as chronic conditions progressed. While stroke and cancer lead to disability immediately, conditions such as diabetes, cancer, and hypertension give rise to higher increments of physical disability in later stage disease. Mitigating depressive symptoms may be beneficial in terms of preventing disability development in this population.

## Background

Over recent decades, the global population of elderly people increased from 5% in 1950 to a projected 16% by 2050 [1] and is aging rapidly, especially in Asian countries, including Taiwan. The ratio of the population aged ≥65 years in Taiwan reached 14% (cut-off point for an “aged” society) in 2018, and estimates are that the ratio will rise to 20% (cut-off point for a “super-aged” society) by 2026. In 2065, older people could account for 41.2% of the entire Taiwanese population [2]. The increase in the number of older people raises concern about a growing strain on healthcare systems.

Chronic disease is a common issue in the elderly population and is the primary health problem in this population, leading to physical disability and even death. In Taiwan, the prevalence rate of at least one chronic disease among elderly people aged over 65 years old is 81.1%, and the prevalence of chronic diseases in elderly people ranks in the following order: hypertension (54.5%), osteoporosis (32.9%), diabetes mellitus (24.7%), heart disease (21.5%), and dyslipidemia (19.6%) [3]. Previous studies have shown that disability is one complication of chronic diseases [4–6] and also accounts for the vast majority of healthcare expenditures [7]. Due to the rapid growth of the elderly population and lifespan elongation, it is worth investigating which chronic diseases lead to greater levels of disability in the elderly population and the associated factors that lead to physical disabilities.

Physical disabilities seriously threaten the independence and quality of life of the elderly population and are generally evaluated according to the ability of individuals to perform activities of daily living (ADLs) [8] and instrumental activities of daily living (IADLs) [9]. Limitations on activities requiring strength and mobility are also regarded as an early sign of frailty and a predictor of later functional disability [10]. Previous studies discussing disability have mainly focused on the measurement of ADLs [11, 12]. Nevertheless, broader evaluations that include difficulties in the IADLs and in strength and mobility can help address the recent trend toward promoting the health of the elderly population [13, 14]. This composite measure makes it possible to assess a broader range of physical disabilities, from preclinical disabilities to later personal care disabilities.

Recent cross-sectional studies have shown that chronic diseases such as arthritis [12, 15], heart disease [12], lung disease [12], hypertension [16, 17], diabetes mellitus [18] and stroke [12] are associated with physical disability in the elderly population. In addition, several longitudinal studies have demonstrated that chronic conditions increase the risk of disability, such as stroke [19], acute myocardial infarction [19], diabetes mellitus [14, 20], chronic obstructive pulmonary disease [21] and arthritis [11]. However, the aforementioned studies mainly explored disability in terms of single diseases with short follow-up intervals and fewer repeated follow-up points. In addition, only one study conducted in the U.S. explored an age-related physical functioning trajectory for several common chronic conditions [13]. However, prevalent and incident diseases could not be differentiated in that study. Also, this study was conducted in a western country, and different country and ethnicity may also lead to different disability trajectories. To the best of our knowledge, there is no study available in eastern countries discussing the trajectory of physical disability after a disease diagnosis among various chronic conditions. So as to fill the current research gaps, the aim of this study is to (1) ascertain the average levels and rates of change in the development of disability after disease diagnosis and (2) determine the influences of sociodemographic and health-related correlates in the development of disability.

## Methods

### Participants

In this study, datasets from the Taiwan Longitudinal Study on Aging (TLSA), an ongoing nationally representative panel survey that collects information on a number of variables related to physical and mental health, living and social arrangement, and retirement planning in middle-aged and older Taiwanese individuals, were used. The initial cohort in 1989 included 4049 participants aged ≥60 years old and re-interviewed every 3–4 years. Later in 1996, another 2462 participants aged 50 to 66 years was supplemented in the original cohort. As a result, the 1996 TLSA sample represented the entire Taiwanese population aged 50 and over living in an institution or in the community (*n* = 5131). A detailed description of the enrollment in the TLSA have been described previously [20]. The Ethical Committee for Human Research at National Cheng Kung University Hospital approved this study (B-ER-104-077). The analytic sample included the data from the 1996, 1999, 2003, 2007, and 2011 interview waves. Self-reported chronic conditions in 1996 and newly diagnosed chronic conditions during follow-ups were included in the final analysis, yielding a sample of 1435 participants with hypertension, 1193 with arthritis, 915 with heart disease, 614 with lung disease, 570 with diabetes mellitus, 277 with stroke, and 192 with cancer.

### Chronic conditions

The chronic conditions’ status was defined by self-reported answers to the dichotomous question “Have you been diagnosed with hypertension, heart disease, stroke, diabetes mellitus, cancer, lung disease, or arthritis by a physician?” Self-reported chronic conditions in 1996 and newly diagnosed chronic conditions during follow-ups until 2011 were collected for each chronic condition.

### Duration after the diagnosis of a chronic condition

The duration after the diagnosis of a chronic condition was recorded for prevalent cases in 1996 and incident cases between 1999 and 2011. For participants who self-reported chronic conditions in 1996, the duration of disease equaled an additional 3 years in 1999, an additional 7 years in 2003, an additional 11 years in 2007, and an additional 15 years in 2011. As for the self-reported newly diagnosed participants between 1999 and 2011, the duration after the diagnosis of a chronic condition was assumed to be 2 years (rounding up 1.5 years in 1999 to the nearest integer) in the reporting wave, and additional 6, 10, 14 years in the following wave, respectively).

### Physical disability scores

Participants in the TLSA cohort were assessed at each wave whether they had any limitation to carry out each of a number of different tasks, including six ADLs (eating, dressing, bathing, toileting, walking across a room, and getting in/out of bed, independently) [8], four IADLs (shopping for personal items, making a phone call, doing light housework, and riding a bus or train) [9], and seven strength and mobility activities (reaching above the head, stooping/ kneeling/or crouching, standing for 15 min, walking 200–300 m, running a short distance (20–30 m), climbing one or two flights of stairs, and lifting or carrying weights over 10 pounds like a heavy bag of groceries) [22]. Physical disability scores were the sum of the 17 dichotomous scores for the ADL, IADL, and strength and mobility activities, where 0 means no difficulties and 1 means some difficulties, and higher scores indicate more severe limitations (range 0–17).

### Other variables

Sociodemographic and health-related covariates that may interfere with physical disability conditions were measured at each wave. Sociodemographic variables included age at initial diagnosis (centered to 70 years old), gender, and education level. Education level was divided into two groups, based on having received formal education and being literate or illiterate. Health-related variables included a number of comorbidities and depression status, and they were modeled as time-varying covariates. The number of comorbidities was defined from a count of selected chronic conditions that the participants self-reported they had been diagnosed with (range 0–6). The 10-item short form of the Centers for Epidemiological Studies-Depression (CES-D) scale [23] scores were measured at each wave (range 0–30). Depression status was divided into two groups according to a CES-D score≧10 or below for the purpose of identifying level of depression [24].

### Statistical analyses

The data were analyzed using SAS software version 9.4 (SAS Institute, Inc., Cary, NC). Pearson’s χ2 tests and *t*-tests were used to compare the differences between the characteristics of participants who suffered from chronic conditions in 1996 and those who did not. *p* < 0.05 was considered to be statistically significant. Because multilevel modeling (MLM) [25] has the capacity to handle the irregular interview interval and control for time-varying covariates, it was used to examine the physical disability trajectories after disease diagnosis and computed different models for each chronic condition. The MLM equation models are shown as follows:

Level-1 model:

$$ {Y}_{ij}={\pi}_{0j}+{\pi}_{1j}{\left( years\ from\ diagnosis\right)}_{ij}+{\pi}_{2j}{{\left( years\ from\ diagnosis\right)}^2}_{ij}+{r}_{ij} $$

where

$$ {r}_{ij}\sim N\left(0,{\sigma}^2\right) $$

Level-2 model:

$$ {\displaystyle \begin{array}{c}{\pi}_{0j}={\beta}_{00}+{\beta}_{01}{COVAR}_j+{u}_{0j,}\\ {}{\pi}_{1j}={\beta}_{10}+{\beta}_{11}{COVAR}_j+{u}_{1j,}\\ {}{\pi}_{2j}={\beta}_{20}+{u}_{2j},\end{array}} $$

where

$$ \left(\begin{array}{c}\begin{array}{c}{u}_{oj}\\ {}{u}_{1j}\end{array}\\ {}{u}_{2j}\end{array}\right)\sim N\left[\left(\begin{array}{c}0\\ {}0\\ {}0\end{array}\right),\left(\begin{array}{ccc}{\tau}_{00}& {\tau}_{01}& {\tau}_{02}\\ {}{\tau}_{10}& {\tau}_{11}& {\tau}_{12}\\ {}{\tau}_{20}& {\tau}_{21}& {\tau}_{22}\end{array}\right)\right]. $$

Initially, we use a model with the intercept, linear, and quadratic growth curve models of physical disability after a chronic condition diagnosis. Then, four models were built within each health outcome. Model 0 represented the independent effect on the levels and rates of change in physical disability among different chronic conditions. The intercept and slope coefficient represented the average level at disease diagnosis and linear rate of change in physical disability after disease diagnosis, respectively. The quadratic term coefficient described an accelerated increase or decrease per year. Model 1 added sociodemographic covariates, including age at initial diagnosis, gender, and education level, with a reference group of males and participants who had received a formal education or were literate. Model 2 added the number of comorbidities treated as time-varying covariates to control for the effect of morbidity. Model 3 added time-varying depression status based on Model 2.

## Results

The baseline characteristics of the participants with and without common chronic conditions in 1996 are shown in Table 1. The number and percentage for each chronic condition in 1996 were 1344 (26.2%) with hypertension, 743 (14.5%) with heart disease, 233 (4.5%) with stroke, 559 (10.9%) with diabetes, 68 (1.3%) with cancer, 497 (9.7%) with lung disease, and 924 (18%) with arthritis, respectively. The participants were older in stroke, heart disease, and lung disease. Participants were predominantly female in hypertension, diabetes, heart disease, and arthritis, whereas more males had been diagnosed with stroke and lung disease. Participants with diabetes, heart disease, and arthritis revealed lower education level. The comorbidity levels were highest for stroke, followed by heart disease and cancer. The highest physical disability scores were 9.44 (SD = 6.56) for stroke, followed by cancer (mean = 4.72, SD = 5.50), heart disease (mean = 3.77, SD = 4.45), lung disease (mean = 3.70, SD = 4.68), diabetes (mean = 3.69, SD = 4.82), arthritis (mean = 3.50, SD = 4.14), and hypertension (mean = 3.28, SD = 4.55).

**Table 1:** Sample sociodemographics of adults with and without the selected seven chronic conditions, TLSA 1996

	Hypertension		Heart disease		Stroke		Diabetes		Cancer		Lung disease		Arthritis	
	No (*n* = 3787)	Yes (*n* = 1344)	No (*n* = 4388)	Yes (*n* = 743)	No (*n* = 4898)	Yes (*n* = 233)	No (*n* = 4572)	Yes (*n* = 559)	No (*n* = 5063)	Yes (*n* = 68)	No (*n* = 4634)	Yes (*n* = 497)	No (*n* = 4207)	Yes (*n* = 924)
Age (years)	**65.50 ± 9.60**	**68.08 ± 8.77**	**65.60 ± 9.55**	**69.58 ± 8.10**	**65.97 ± 9.47**	**70.44 ± 7.96**	**66.07 ± 9.59**	**67.05 ± 8.24**	66.17 ± 9.45	66.94 ± 9.59	**65.82 ± 9.45**	**69.55 ± 8.84**	**65.74 ± 9.51**	**68.19 ± 8.93**
Age														
50–64	**45.8**	**32.2**	**45.3**	**24.2**	**43.2**	**21.5**	**42.9**	**37.0**	42.3	36.8	**44.1**	**25.4**	**44.6**	**31.6**
65–74	**35.9**	**45.9**	**36.5**	**50.5**	**38.0**	**49.8**	**37.7**	**45.6**	38.5	44.1	**37.8**	**45.3**	**36.9**	**46.1**
≧75	**18.3**	**21.9**	**18.2**	**25.3**	**18.7**	**28.8**	**19.4**	**17.4**	19.2	19.1	**18.1**	**29.4**	**18.5**	**22.3**
Gender														
Men	**55.5**	**49.1**	**55.1**	**46.0**	**53.2**	**66.1**	**54.6**	**47.4**	53.8	55.9	**52.5**	**65.79**	**56.8**	**40.3**
Women	**44.6**	**50.9**	**44.9**	**54.0**	**46.8**	**33.9**	**45.4**	**52.6**	46.2	44.1	**47.5**	**34.21**	**43.2**	**59.7**
Education status														
Without education	32.3	33.6	**31.5**	**39.0**	32.6	33.5	**32.1**	**36.7**	32.6	30.9	32.8	30.8	**31.0**	**39.8**
With education	67.7	66.4	**68.5**	**61.0**	67.4	66.5	**67.9**	**63.3**	67.4	69.1	67.2	69.2	**69.0**	**60.2**
Number of comorbidities	**0.46 ± 0.71**	**0.96 ± 0.98**	**0.60 ± 0.78**	**1.33 ± 1.13**	**0.77 ± 0.95**	**1.55 ± 1.15**	**0.70 ± 0.90**	**1.10 ± 1.11**	**0.83 ± 1.01**	**1.22 ± 1.23**	**0.71 ± 0.91**	**1.13 ± 1.15**	**0.61 ± 0.85**	**0.95 ± 1.03**
Depression status														
Without depression	**79.5**	**73.1**	**80.0**	**64.9**	**78.6**	**57.1**	**78.9**	**69.0**	**78.0**	**61.0**	**79.5**	**61.3**	**80.6**	**65.7**
With depression	**20.5**	**26.9**	**20.0**	**35.1**	**21.4**	**42.9**	**21.1**	**31.1**	**22.0**	**39.0**	**20.5**	**38.7**	**19.5**	**34.3**
Physical disability score	**1.81 ± 3.47**	**3.28 ± 4.55**	**1.93 ± 3.66**	**3.77 ± 4.45**	**1.85 ± 3.28**	**9.44 ± 6.56**	**2.01 ± 3.66**	**3.69 ± 4.82**	**2.16 ± 3.80**	**4.72 ± 5.50**	**2.03 ± 3.70**	**3.70 ± 4.68**	**1.91 ± 3.71**	**3.50 ± 4.14**
Disease duration (years)		7.50 ± 7.41		7.65 ± 8.11		5.81 ± 5.63		7.86 ± 7.20		5.85 ± 8.27		10.02 ± 11.80		8.61 ± 8.92

Data are given as either % or means ± SD. Bold numbers indicate statistical significance (*p* < 0.05)

Table 2 shows the changes in mean physical disability scores related to years after diagnosis. The results revealed that there was an increasing growth pattern in physical disability after disease diagnosis, which means that it was appropriate to use multilevel modeling method to measure physical disability.

**Table 2:** Raw mean scores and standard deviations of physical disability development of participants with different chronic conditions

	Hypertension	Heart disease	Stroke	Diabetes	Cancer	Lung disease	Arthritis
Before diagnosis	1.86 ± 3.08	2.18 ± 3.29	2.87 ± 3.81	2.42 ± 3.65	2.03 ± 3.19	2.69 ± 3.90	2.10 ± 3.25
2 years after diagnosis	3.89 ± 4.98	4.66 ± 5.06	9.77 ± 6.31	4.83 ± 5.38	5.43 ± 5.57	5.08 ± 5.50	4.27 ± 4.67
6 years after diagnosis	4.28 ± 5.09	4.93 ± 5.19	9.22 ± 6.27	4.91 ± 5.55	4.28 ± 4.76	5.12 ± 5.54	4.64 ± 4.90
10 years after diagnosis	4.76 ± 5.47	5.52 ± 5.38	9.46 ± 6.42	5.75 ± 5.80	4.89 ± 5.28	5.34 ± 5.57	5.32 ± 5.26
14 years after diagnosis	5.52 ± 5.81	6.13 ± 5.66	8.86 ± 6.23	6.20 ± 6.30	5.63 ± 5.48	6.02 ± 5.84	5.49 ± 5.44

**Fig. 1:**
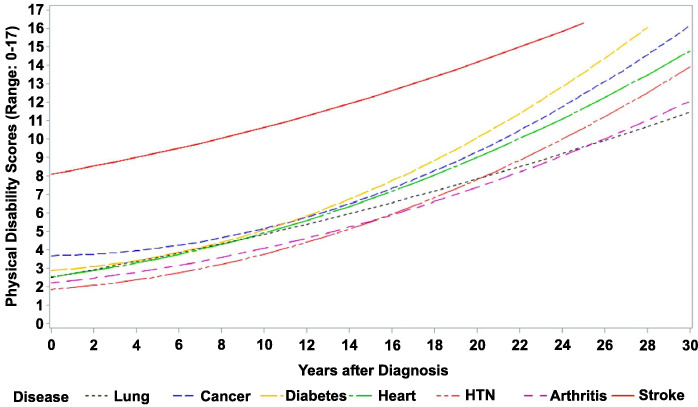
Physical disability trajectories after diagnosis for each chronic condition in adults over 50 years of age (TLSA 1996–2011)

Table 3 shows the physical disability trajectories for participants with different newly diagnosed chronic conditions over time. In Model 0, the estimated intercept, linear, and quadratic changes in physical disability without controlling for any covariates were determined, for which the mean growth trajectories of physical disability are shown in Fig. 1. We found that (1) the physical disability was highest for stroke (β_stroke_ = 8.104, 95% CI = 7.203–9.004), followed by cancer (β_cancer_ = 3.693, 95% CI = 2.880–4.506) and diabetes (β_diabetes_ = 2.887, 95% CI = 2.442–3.332) at initial disease diagnosis. (2) The linear rate of change in physical disability after disease diagnosis was highest for stroke (β_stroke*time_ = 0.203, 95% CI = 0.041–0.364), followed by lung disease (β_lung disease*time_ = 0.200, 95% CI = 0.144–0.257) and heart disease (β_heart disease*time_ = 0.155, 95% CI = 0.102–0.207), indicating that these diseases led to a higher steady increase in physical disability after disease diagnosis. (3) The quadratic rate of change in physical disability after disease diagnosis was highest for diabetes (β_diabetes*time_^2^ = 0.014, 95% CI = 0.010–0.017), followed by cancer (β_cancer*time_^2^ = 0.013, 95% CI = 0.007–0.020) and hypertension (β_hypertension*time_^2^ = 0.010, 95% CI = 0.009–0.012), indicating that these diseases exhibited higher increments of physical disability at later stages of disease. Furthermore, we also determined the estimated changes in physical disability at 5-year intervals after diagnosis of each chronic condition, for which the results are presented in Table 4.

**Table 3:** Levels and rates of change in the physical disability trajectory after diagnosis for each chronic condition

		Fixed effect			Goodness of fit		
		Intercept	Linear change	Quadratic change	-2LL	Parameters	LR test △χ^2^ (△df)
Hypertension	Model 0	**1.858 (1.615–2.101)**	**0.087 (0.046–0.129)**	**0.010 (0.009–0.012)**	25,401.7	7	
	Model 1	**1.028 (0.735–1.320)**	**0.174 (0.132–0.216)**	**0.007 (0.005–0.008)**	24,851.0	12	550.7 (5)***
	Model 2	**0.441 (0.150–0.731)**	**0.154 (0.113–0.196)**	**0.006 (0.005–0.008)**	24,591.9	13	259.1 (1)***
	Model 3	**0.271 (0.030–0.512)**	**0.120 (0.085–0.156)**	**0.004 (0.003–0.006)**	21,169.2	15	3422.7 (2)***
Heart disease	Model 0	**2.538 (2.223–2.853)**	**0.155 (0.102–0.207)**	**0.008 (0.006–0.010)**	16,068.7	7	
	Model 1	**1.358 (0.959–1.756)**	**0.235 (0.183–0.288)**	**0.004 (0.002–0.007)**	15,684.4	12	384.3 (5)***
	Model 2	**0.425 (0.009–0.841)**	**0.222 (0.170–0.274)**	**0.004 (0.002–0.006)**	15,563.0	13	121.4 (1)***
	Model 3	**0.558 (0.208–0.908)**	**0.161 (0.117–0.205)**	**0.003 (0.001–0.004)**	13,317.3	15	2245.7 (2)***
Stroke	Model 0	**8.104 (7.203–9.004)**	**0.203 (0.041–0.364)**	0.005(−0.004–0.014)	3632.9	7	
	Model 1	**5.104 (4.040–6.169)**	**0.288 (0.129–0.447)**	0.001(− 0.007–0.010)	3510.6	12	122.3 (5)***
	Model 2	**4.509 (3.293–5.724)**	**0.289 (0.130–0.449)**	0.001(−0.008–0.009)	3506.7	13	3.9 (1)*
	Model 3	**3.018 (1.861–4.175)**	**0.215 (0.054–0.376)**	0.002(− 0.007–0.010)	2495.0	15	1011.7 (2)***
Diabetes	Model 0	**2.887 (2.442–3.332)**	0.080(−0.001–0.161)	**0.014 (0.010–0.017)**	9469.6	7	
	Model 1	**2.209 (1.676–2.742)**	**0.173 (0.091–0.255)**	**0.009 (0.005–0.012)**	9207.5	12	262.1 (5)***
	Model 2	**1.080 (0.517–1.643)**	**0.166 (0.086–0.247)**	**0.008 (0.004–0.011)**	9102.6	13	104.9 (1)***
	Model 3	**0.805 (0.331–1.278)**	**0.105 (0.037–0.172)**	**0.006 (0.003–0.009)**	7680.5	15	1422.1 (2)***
Cancer	Model 0	**3.693 (2.880–4.506)**	0.013(−0.137–0.163)	**0.013 (0.007–0.020)**	2554.0	7	
	Model 1	**2.053 (1.079–3.027)**	0.133(−0.015–0.282)	**0.009 (0.002–0.016)**	2467.9	12	86.1 (5)***
	Model 2	**1.156 (0.172–2.141)**	0.088(−0.063–0.238)	**0.009 (0.003–0.016)**	2442.1	13	25.8 (1)***
	Model 3	0.542(−0.184–1.268)	0.079(− 0.041–0.198)	**0.007 (0.001–0.012)**	2011.6	15	430.5 (2)***
Lung disease	Model 0	**2.526 (2.100–2.953)**	**0.200 (0.144–0.257)**	**0.003 (0.002–0.005)**	10,865.3	7	
	Model 1	**1.336 (0.863–1.810)**	**0.305 (0.251–0.360)**	−0.0004(−0.002–0.001)	10,486.0	12	379.3 (5)***
	Model 2	**0.681 (0.192–1.170)**	**0.291 (0.236–0.345)**	−0.001(− 0.003–0.001)	10,427.1	13	58.9 (1)***
	Model 3	0.366(−0.017–0.750)	**0.213 (0.175–0.250)**	**− 0.001(− 0.002 to − 0.0003)**	8713.5	15	1713.6 (2)***
Arthritis	Model 0	**2.205 (1.942–2.468)**	**0.119 (0.077–0.161)**	**0.007 (0.005–0.008)**	22,100.0	7	
	Model 1	**1.466 (1.132–1.800)**	**0.226 (0.184–0.268)**	**0.003 (0.002–0.005)**	21,496.9	12	603.1 (5)***
	Model 2	**0.833 (0.492–1.174)**	**0.204 (0.162–0.245)**	**0.003 (0.001–0.004)**	21,353.7	13	143.2 (1)***
	Model 3	**0.613 (0.322–0.903)**	**0.183 (0.146–0.219)**	0.001(−0.001–0.002)	18,683.7	15	2670.0 (2)***

Scores of physical disability are the total of limitations in ADL, IADL, and mobility (range 0–17)

Bold numbers and *indicate statistical significance (*p* < 0.05), ***indicate statistical significance (*p* < 0.001)

Model 0: Duration after diagnosis and (duration after diagnosis)^2^

Model 1: Model 0 + age at diagnosis (centered to 70 years old), gender, education level

Model 2: Model 1 + number of comorbidities over time

Model 3: Model 2 + depression status over time

**Table 4:** Disease-related disability burden based on fixed effect coefficients for Model 0

	Hypertension	Heart disease	Stroke	Diabetes	Cancer	Lung disease	Arthritis
Initial diagnosis	1.858	2.538	8.104	2.887	3.693	2.526	2.205
5 years	2.543	3.513	9.244	3.637	4.083	3.601	2.975
10 years	3.728	4.888	10.634	5.087	5.123	4.826	4.095
15 years	5.413	6.663	12.274	7.237	6.813	6.201	5.565
20 years	7.598	8.838	14.164	10.087	9.153	7.726	7.385
25 years	11.283	12.213	16.804	15.037	13.443	9.701	10.255

Model 1 added sociodemographic variables, including age at diagnosis, gender, and education level to determine the impact of these variables on physical disability. These models all significantly improved the model fit from Model 0, indicating that the aforementioned variables were significant predictors of interindividual variations in the physical disability trajectories among the chronic conditions under consideration, accounting for 20.3–35.2% of the variances changes in the mean physical disability intercept and 17.5–41.4% of the variances changes in physical disability over time for different chronic conditions, respectively, for which the detailed results are shown in Table 5. After controlling for sociodemographic variables, participants with stroke (β_stroke_ = 5.104, 95% CI = 4.040–6.169), diabetes (β_diabetes_ = 2.209, 95% CI = 1.676–2.742), and cancer (β_cancer_ = 2.053, 95% CI = 1.079–3.027) still had higher physical disability scores at the initial disease diagnosis. This result indicated that these diseases are independent of sociodemographic characteristics. For participants with lung disease, the significant quadratic rate of change disappeared after controlling for sociodemographic variables, suggesting that sociodemographic covariates play a crucial role in lung disease.

**Table 5:** Comparison of random effects of the different chronic conditions on the nested models

		Variance of intercept (1,1)	Percentage of variance change from previous models	Variance of slope (1,1)	Percentage of variance change from previous models
Hypertension	Model 0	8.516		0.105	
	Model 1	6.664	21.7	0.084	20.0
	Model 2	5.968	10.4	0.081	3.6
	Model 3	3.208	46.2	0.043	46.9
Heart disease	Model 0	10.310		0.102	
	Model 1	8.214	20.3	0.077	24.5
	Model 2	7.251	11.7	0.074	3.9
	Model 3	4.355	39.9	0.041	44.6
Stroke	Model 0	38.806		0.097	
	Model 1	26.718	31.1	0.080	17.5
	Model 2	26.718	0.0	0.087	−8.8
	Model 3	15.980	40.2	0.054	37.9
Diabetes	Model 0	14.373		0.150	
	Model 1	11.386	20.8	0.121	19.3
	Model 2	10.880	4.4	0.118	2.5
	Model 3	6.172	43.3	0.053	55.1
Cancer	Model 0	18.385		0.146	
	Model 1	12.678	31.0	0.119	18.5
	Model 2	11.063	12.7	0.127	−6.7
	Model 3	2.925	73.6	0.055	56.7
Lung disease	Model 0	15.855		0.111	
	Model 1	10.276	35.2	0.065	41.4
	Model 2	9.553	7.0	0.061	6.2
	Model 3	3.316	65.3	0.000	100.0
Arthritis	Model 0	7.907		0.084	
	Model 1	5.965	24.6	0.059	29.8
	Model 2	5.495	7.9	0.056	5.1
	Model 3	2.575	53.1	0.025	55.4

Model 0: Duration after diagnosis and (duration after diagnosis)^2^

Model 1: Model 0 + age at diagnosis (centered to 70 years old), gender, education level

Model 2: Model 1 + number of comorbidities over time

Model 3: Model 2 + depression status over time

Model 2 represented the influence of each chronic condition on physical disability while controlling for sociodemographic variables and time-varying comorbidities. The significance of linear and quadratic rates of change in physical disability in chronic conditions remained unchanged from Model 1 to Model 2 and accounted for 0–12.7% of the changes in the variances in the mean physical disability intercept and − 8.8 to 6.2% of the changes in the variances in the physical disability over time for different chronic conditions. The negative value obtained for the changes in variance between the models for stroke and cancer indicated that comorbidity plays no role in physical disability in the case of these two diseases. After controlling for the above referenced covariates, participants with stroke (β_stroke_ = 4.509, 95% CI = 3.293–5.724), cancer (β_cancer_ = 1.156, 95% CI = 0.172–3.027), and diabetes (β_diabetes_ = 1.080, 95% CI = 0.517–1.643) retained higher physical disability scores at the initial disease diagnosis.

Model 3 represented the influence of each chronic condition on physical disability after controlling for sociodemographic variables, time-varying comorbidities, and depression status. The changes in the variances from Model 2 to Model 3 accounted for 39.9–73.6% of the mean physical disability intercept and 37.9–100% of physical disability over time for different chronic conditions, suggesting that depression status was a robust factor in all chronic conditions. After controlling for the covariates discussed earlier, participants with stroke (β_stroke_ = 3.018, 95% CI = 1.861–4.175), diabetes (β_diabetes_ = 0.805, 95% CI = 0.331–1.278), and arthritis (β_arthritis_ = 0.613, 95% CI = 0.322–0.903) retained higher physical disability scores at the initial disease diagnosis.

## Discussion

To the best of our knowledge, this is the first long-term, population-based nationally representative study to compare the physical disability trajectories after common chronic conditions diagnosis in middle-aged and older adults in the Asian population. Among the seven chronic conditions under consideration, the results showed that (1) at the initial disease diagnosis, physical disability was highest for stroke, followed by cancer and diabetes. (2) The linear rate of change was highest for stroke, followed by lung disease and heart disease. (3) The quadratic rate of change was highest in diabetes, followed by cancer and hypertension, indicating that these diseases showed higher increments of physical disability in later stage disease.

Prior literature comparing multiple chronic conditions has shown that stroke is the most disabling disease [13, 26, 27], which is consistent with our findings. However, an examination of disability in other diseases led to inconsistent results. Previous studies have shown that stroke, treated diabetes, chronic airway obstruction, coronary heart disease, and treated hypertension are all significantly associated with onset of disability [26]. Another study showed newly occurring conditions such as stroke, followed by cancer, heart attack, and diabetes followed up for 6 years led to greater relative risks for developing incident disability in ADL [27]. In addition, one cohort study found that stroke, followed by pulmonary diseases and arthritis, was associated with greater physical functioning difficulties compared with heart disease, cancer, and diabetes [13]. The inconsistency of disabilities in disease rankings among the aforementioned studies may be related to different disability outcome measurements, different study designs, and different countries and ethnicities. The current work provided evidence of average levels and rates of change in physical disability after chronic condition diagnoses in an Asian population.

Previous studies have shown that sociodemographic variables are associated with disabilities, including age, gender, marital status, education, and economic status. Disability has been shown to be more common in females and in divorced/separated/widowed respondents, and has been positively associated with increasing age and inversely associated with education and economic status [28, 29]. In the current study, subjects who were older at the initial diagnosis, female, who had lower educational levels had higher levels of physical disability for all chronic conditions, which was consistent with the findings of prior studies [28, 29].

Multiple existing studies have shown that multimorbidity is associated with disability after controlling for sociodemographic status [13, 17, 30, 31]. In the present study, comorbidity explained small parts of the variance after controlling for sociodemographic covariates. It accounted for 4.4–12.7% of the changes in the variances in the intercept and 2.5–6.2% of the changes in the variances in physical disability over time in chronic conditions, with the exception of stroke and cancer. The influences of comorbidity on disability for stroke were inconsistent. In Stenholm’s study, comorbidity was associated with a greater burden of physical functioning difficulties, and stroke alone or in combination with other diseases is one of the diseases leading to the most limitations in terms of physical functioning [13]. However, in Lopez-Espuela’s study, comorbid conditions did not affect the patients’ functional status 6 months after they had experienced a stroke [32], which was similar to our results. Further detailed exploration discussing the discrepancies in comorbidity on disability related to stroke is therefore needed.

Depression associated with disability is well-established in prior literature in the general elderly population [27, 33], with one study reporting that depression increases the risk of subsequent ADL disability and mobility disability by 67 and 73%, respectively [27]. In addition, one study targeting the diabetes population showed disability to be greater in diabetes patients with depressive symptoms [34]. Indeed, there’s a wide spectrum of opinions on the mechanism for the causal relationship between disability and depression. Some studies have clearly shown that medical illness and physical disability are strongly associated with depression [35, 36]. And the association between disability and increased prevalence of depression irrespective of physical health problems was tested and result showed all ADL/IADL limitations are significantly associated with depression [36]. On the other hand, one cohort study has shown that depression in initial non-disabled older persons significantly increases the risk for subsequent incident ADL and mobility disability [27]. The explanation for depression leading to disability in that study is that lower levels of physical activity and fewer contacts with relatives among depressed persons. Thus, the causal relationship between disability and depression is bi-directional depending on different clinical situations. In our study, time-varying depression status explained a great amount of the variance after controlling for the aforementioned covariates, with 39.9–73.6% changes in the variances in the intercept and 37.9–100% of the changes in the variances in physical disability over time, which was in line with previous findings. The results of this study suggest that depression status may have an impact on physical disability and that intervention for depression management may have a protective effect against the trend in increased physical disability.

This study has some limitations. First, self-reported information on chronic conditions and physical function were used in this national cohort database. The reporting and recall bias from self-reported information may lead to underestimation of disease prevalence, and thus, the results should be interpreted with caution. However, previous studies have shown high levels of accuracy between self-reported data and clinical diagnoses for diseases such as hypertension and diabetes and fair accuracy for lung disease, cardiac disease, stroke, and malignancies, with the exception of arthritis [37–39]. Future research including formal medical records on disease status is encouraged to minimize such biases. Second, the TLSA database does not include annual interviews with the respondents, so we could only record physical disability scores at 3 to 4 year intervals to estimate the trajectories. Thus, subtle between-interval changes in physical disability may have been missed. Also, there may have been a survivorship bias due to the increasing mortality associated with the duration of the disease. Third, because the present study was aimed toward describing development of disability after a diagnosis of different chronic conditions, the time axis was fixed to years after diagnosis. Thus, a comparison of the disability burden related to each chronic condition with the normal aging process could not be achieved via this study. In future studies, researchers are encouraged to use age as a time axis to distinguish the burden of each chronic condition as compared with normal aging [20]. Finally, due to the limited TLSA interview data, we were unable to distinguish participants’ economic status and further chronic condition details, such as disease type, severity and self-management status. The current work could only depict the mean physical disability trajectory, so future studies with detailed disease status information may help clarify their contribution to the physical disability trajectory. Also, common mental diseases such as depression and memory-related diseases were not included in our self-reported chronic condition questionnaire, so future studies could include such diseases simultaneously for comparison.

## Conclusions

Despite the fact that a comparison across conditions was not statistically tested, an accelerated increase in physical disabilities was found as chronic conditions progressed. While stroke and cancer lead to disability immediately, conditions such as diabetes, cancer, and hypertension give rise to higher increments of physical disability in later stages of disease. Mitigating depressive symptoms may be beneficial in terms of preventing disability development in this population.

## Data Availability

The data that support the findings of this study are available from Health Data Science Center, National Cheng Kung University Hospital, but restrictions apply to the availability of these data, which were used under license for the current study and are thus not publicly available. Data are, however, available from the authors upon reasonable request and with permission of the Health Data Science Center, National Cheng Kung University Hospital.

## Abbreviations

ADL: Activities of Daily Living
IADL: Instrumental Activities of Daily Living
TLSA: Taiwan Longitudinal Study on Aging
CES-D: Centers for Epidemiological Studies-Depression
MLM: Multilevel Modeling
SD: Standard Deviation
CI: Confidence Interval
